# A maximum likelihood estimator of neural network synaptic weights

**DOI:** 10.1186/1471-2202-14-S1-P59

**Published:** 2013-07-08

**Authors:** Wahiba Taouali, Bruno Cessac

**Affiliations:** 1 NeuroMathComp team (INRIA, UNSA LJAD), Sophia Antipolis, France

## 

The statistics of spikes in a neuronal network is constrained on one hand by the stimulus and shared noise, and on the other hand by neuron interactions and collective dynamics. The join spike statistics and its spatio-temporal correlations can be explicitly computed in conductance-based Integrate-and-Fire models [1,2]. The probability distribution of spike is a Gibbs distribution (in its most general definition allowing to consider non-stationarity) which encompasses existing statistical models such as Maximum Entropy models or Generalized-Linear Models.

Moreover, the dependence of spike statistics in network parameters such as synaptic weights and stimulus is explicit.

**Figure 1:**
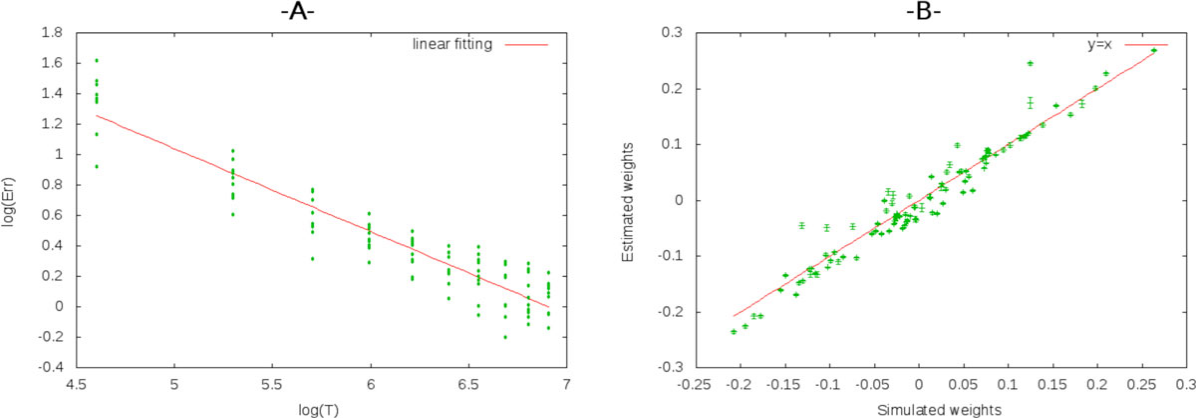
**A: The mean percentage error is calculated over the estimated weights of 10 fully connected neurons (100 weights) using for each point a different bloc raster of same or different sizes**. B. The error bars correspond to the variation of the estimated weights function of the real weights for an observation time T = 500.

Here, we address the following reverse engineering problem. Given a conductance-based Integrate-and-Fire model as above where the spike statistics dependence on synaptic weights is known, can one reconstruct this network of synaptic weights from the observation of a raster plot generated by the network ? We have solved this inverse problem using an explicit expression of a maximum likelihood estimator based on the Newton-Raphson method. This estimator employs analytically computed gradients and Hessian of the likelihood function given by the product of conditional probabilities. The explicit form of these conditional probabilities can be found in [1]. Our results show that this method allows to estimate the set of connections weights knowing the input, the noise distribution and the leak function. Moreover, we found that, in a log scale scheme, the estimation mean percentage error Err decreases linearly with observation time T (Figure 1).

This estimator is based on a plausible probabilistic model of spiking activity, and not a Poisson likelihood processing. So, it offers a flexible framework that should allow better statistical analysis of real data.
